# The expanding landscape of adipokines: emerging roles of PAI-1 and vaspin in cardiometabolic diseases

**DOI:** 10.3389/fendo.2026.1787458

**Published:** 2026-03-26

**Authors:** Sule Kocabas, Nevin Sanlier

**Affiliations:** Department of Nutrition and Dietetics, School of Health Sciences, Ankara Medipol University, Altındağ, Ankara, Türkiye

**Keywords:** cardiovascular diseases, diabetes, metabolic syndrome, obesity, PAI-1, vaspin

## Abstract

Cardiometabolic diseases are chronic conditions arising from the common pathophysiology of metabolic and cardiovascular disorders accompanied by risk factors such as insulin resistance, obesity, type 2 diabetes, metabolic syndrome, and hypertension. In recent years, the relationship between adipokines such as PAI-1 and vaspin and these diseases has attracted increasing interest. PAI-1 increases cardiovascular risks by inhibiting fibrinolysis, and high PAI-1 levels are associated with obesity and insulin resistance. Vaspin, on the other hand, may have an inhibitory effect on the development of type 2 diabetes and metabolic syndrome by increasing insulin sensitivity. Considering the effects of dietary and lifestyle factors on these molecules, PAI-1 and vaspin are thought to have potential as early biomarkers and therapeutic targets. However, conflicting findings in the literature necessitate further research. Alongside lifestyle interventions based on healthy eating and exercise, changes in PAI-1 and vaspin levels show promise as potential biomarkers for the early diagnosis and prevention of cardiometabolic disorders and the development of personalized treatment strategies. Further research is required to better clarify the molecular mechanisms regulating PAI-1 and vaspin and to determine their potential clinical applications in the prevention and management of cardiometabolic diseases.

## Graphical Abstract

**Figure:**
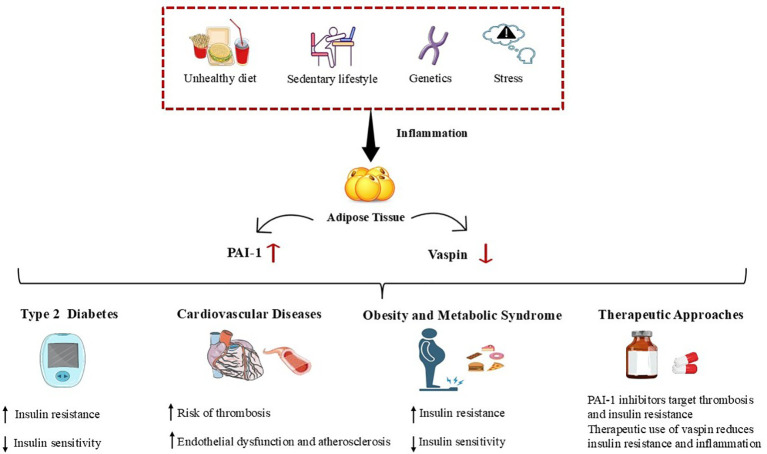

## 1 Introduction

Obesity, type 2 diabetes (T2DM), insulin resistance, fatty liver disease, and cardiovascular diseases are categorized as cardiometabolic diseases and pose a growing threat worldwide (1). Such processes can also be understood as composing a spectrum of interconnected pathophysiological changes that significantly increase the risk of disorders of the cardiovascular system and related metabolic organs (2). Several well-known genetic and environmental risk factors are associated with cardiometabolic diseases, such as smoking, abdominal obesity, insulin resistance, high blood pressure, high cholesterol, unhealthy diet, and socioeconomic status (SES) (3). Individuals with lower socioeconomic status often have limited access to healthy foods, safe environments for physical activity, and health education, which may increase the risk of obesity and related metabolic disorders. In many high- and middle-income countries, lower SES has been consistently associated with higher prevalence of obesity and unhealthy lifestyle behaviors (4, 5). Interestingly, the prevalence of these cardiometabolic features is increasing steadily. Growing evidence indicates that obesity-related inflammation may have a significant role in the pathogenesis of such disorders. Obesity triggers chronic low-grade inflammation through the increased expression of proinflammatory genes and heightened oxidative stress (6, 7). These inflammatory processes play a key role in the onset and progression of cardiometabolic diseases such as T2DM, cardiovascular diseases, and metabolic syndrome (MetS) associated with obesity (8). Understanding these interrelationships is crucial for effective prevention and treatment strategies.

Adipose tissue has long been recognized as an active endocrine organ that secretes a variety of adipokines regulating metabolic and inflammatory processes. Among these molecules, leptin and adiponectin are considered the most extensively studied and historically important adipokines. Leptin, first identified in 1994, plays a central role in regulating energy balance, appetite control, and neuroendocrine signaling, and elevated leptin levels are often associated with obesity and leptin resistance (9, 10). In contrast, adiponectin is known for its insulin-sensitizing, anti-inflammatory, and anti-atherogenic effects, and reduced circulating adiponectin levels are strongly linked to insulin resistance, type 2 diabetes, and cardiovascular diseases (11, 12). These adipokines have provided fundamental insights into the endocrine function of adipose tissue and have paved the way for the identification of additional adipose-derived mediators such as PAI-1 and vaspin. Adipose tissue, a dynamic endocrine organ, secretes various bioactive molecules known as adipokines, which play important roles in metabolic homeostasis and cardiovascular health. Adipokines help to regulate a wide variety of biological processes including insulin resistance, inflammation, and lipid and glucose metabolism (13). Plasminogen activator inhibitor-1 (PAI-1) and visceral adipocyte-derived serine protease inhibitor (vaspin) are key regulatory molecules in the pathophysiology of metabolic disorders and play important roles in obesity-related metabolic diseases, T2DM, MetS, and cardiovascular diseases (14, 15). PAI-1, primarily produced by fat cells, is a serine protease inhibitor that contributes to insulin resistance, endothelial dysfunction, and increased thrombotic risk, thereby exacerbating the progression of T2DM, obesity, and cardiovascular diseases. PAI-1 is intensively studied as a multisystemic biomarker and therapeutic target due to its involvement in multidimensional biological processes such as inflammation, thrombosis, metabolic disorders, and obesity, particularly via its increased expression in visceral adipose tissue (16, 17).

Vaspin is an adipokine that stands out for its anti-inflammatory effects, insulin-sensitizing properties, and potential protective impact on the cardiovascular system. Due to these multifaceted biological effects, vaspin has been increasingly studied in recent years in relation to the pathophysiology of T2DM, obesity, MetS, and cardiovascular diseases (16, 18). Understanding the complex mechanisms and signaling pathways governing interactions between PAI-1 and vaspin is crucial in efforts to clarify the intricate relationship between adipose tissue dysfunction and the development of T2DM, obesity, and associated cardiovascular risks.

This review examines the relationships between levels of PAI-1 and vaspin, which are prominent biomarkers and potential therapeutic targets associated with cardiometabolic diseases, and the pathophysiological mechanisms of action of these adipokines. It also examines current scientific evidence and discussions regarding the association of these adipokines with processes such as inflammation, insulin resistance, obesity, and vascular dysfunction. This study aims to make a synthesizing contribution to the scattered body of knowledge in the literature by evaluating PAI-1 and vaspin together. It is also anticipated that this study will make an original holistic contribution to the literature on cardiometabolic disease by comprehensively examining these adipokines in terms of secreting tissues, receptor-level pathways, relationships with diet and lifestyle factors, potential for clinical use, and roles in prevention and treatment strategies.

## 2 Structure, pathophysiology, and mechanism of action of PAI-1

PAI-1 is a 50-kDa single-chain glycoprotein belonging to the serine protease inhibitor (serpin) superfamily (19). PAI-1 exists in multiple conformational states, including active, latent, and cleaved forms, which critically determine its biological activity. The active conformation can form inhibitory complexes with tissue plasminogen activator (tPA) and urokinase-type plasminogen activator (uPA), thereby suppressing plasmin formation and fibrinolysis (20, 21). However, active PAI-1 is structurally unstable and can spontaneously convert to the latent form by the addition of the reactive center ring to the central β-sheet of the serpin structure. This conformational change prevents the molecule from inhibiting target proteases and represents an important regulatory mechanism that controls the fibrinolytic balance under physiological and pathological conditions (20, 21). The gene that encodes PAI-1 in humans is located on chromosome 7 and spans the q21.3 band, comprising 9 exons and 8 introns and covering approximately 12.2 kb of DNA. Based on NH2-terminal heterogeneity, it consists of either 379 or 381 amino acids that do not contain cysteine (19, 22). PAI-1 is a protein that plays an important role in regulating fibrinolysis, the process by which blood clots are broken down in the body. It is considered the main fibrinolysis regulator due to its rapid inhibition of tissue plasminogen activator (tPA) and urokinase-type plasminogen activator (uPA) (23). In this way, PAI-1 regulates fibrinolysis and the remodeling of the extracellular matrix (ECM) (24). An important regulator of PAI-1 stability and function is its interaction with vitronectin, an extracellular matrix glycoprotein found in plasma and vascular tissues. PAI-1 binding to vitronectin stabilizes the inhibitor’s active conformation and prolongs its functional half-life in circulation (20, 25). The PAI-1-vitronectin complex also influences vascular remodeling, inflammation, and tissue repair processes by regulating integrin-dependent cell adhesion and migration. Through these mechanisms, the interaction between PAI-1 and vitronectin contributes to the broader roles of PAI-1 in thrombosis, fibrosis, and the progression of cardiometabolic diseases (20, 25). With mediation by tPA, plasmin contributes to fibrinolysis because it breaks down the insoluble fibrin network that forms blood clots (26). The function of the plasminogen/plasmin system via uPA-mediated plasminogen activation extends to pericellular proteolysis, which is associated with processes such as tissue remodeling and cell migration (26). More recent studies have identified additional tPA targets capable of contributing to cardiovascular aging processes, such as brain-derived neurotrophic factor (BDNF), fibroblast growth factor 23 (FGF23), matrix metalloproteinase-9 (MMP-9), and insulin-like growth factor-binding protein 3 (IGFBP3) (24, 27). It has been demonstrated that PAI-1’s inhibition of tPA and uPA may foster ECM protein accumulation and the exacerbation of vascular stiffness. The uPA/tPA/plasmin/MMP proteolytic system is responsible for regulating ECM remodeling and homeostasis (17, 26, 28). PAI-1 serves as an upstream inhibitor of uPA and tPA, thereby affecting plasmin and MMP activities as targets of tPA and uPA. Thus, it disrupts various components of the ECM (28). Elevated PAI-1 levels consequently play a role in tissue fibrosis driven by reduced ECM degradation and altered cellular migration (24). Furthermore, it has been suggested that PAI-1 binds directly to endothelial nitric oxide synthase (eNOS) and inhibits eNOS activity in cultured endothelial cells. For this reason, eNOS inhibition by PAI-1 may increase intracellular reactive oxygen species (ROS) (29).

**Figure 1:**
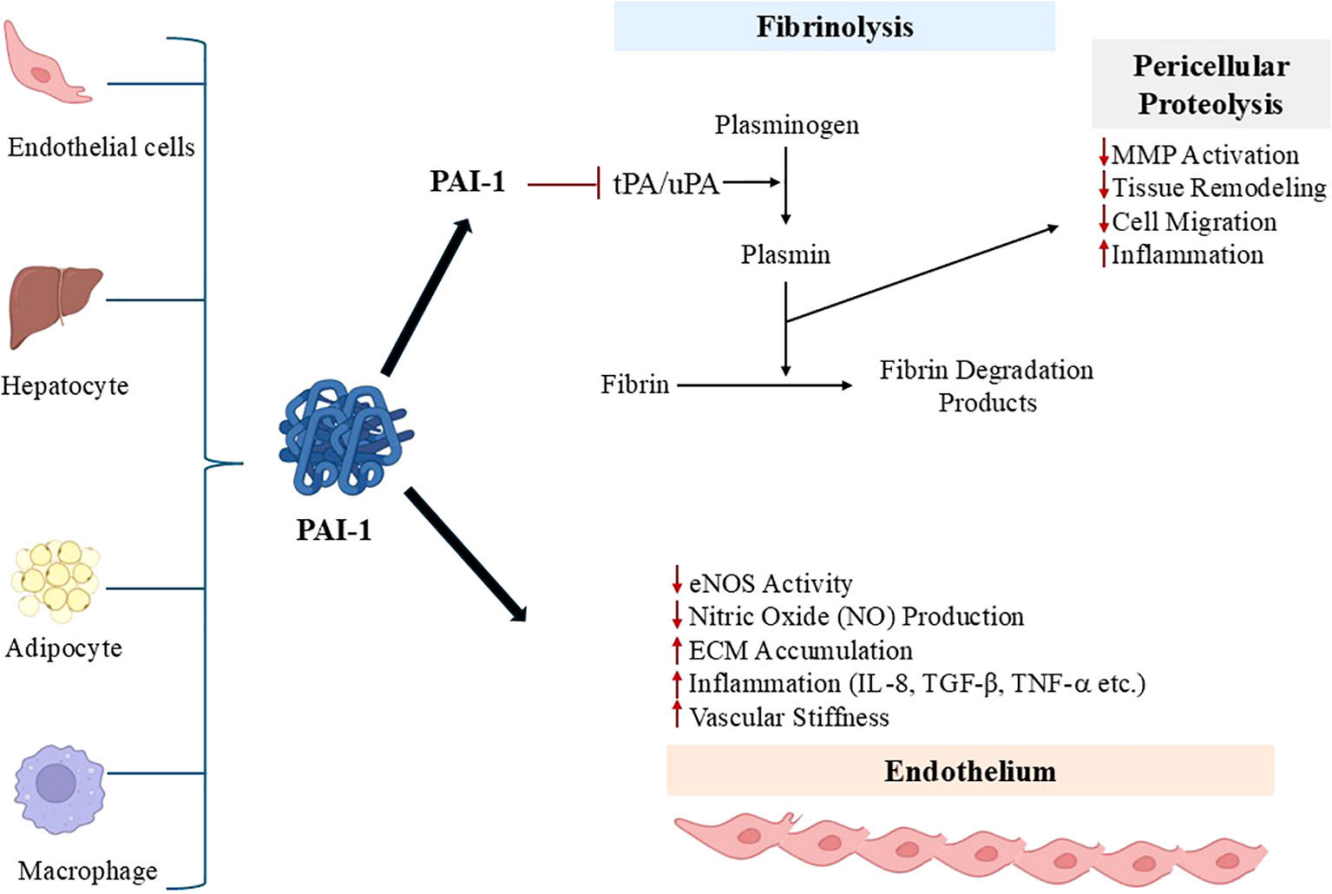
Cellular sources of PAI-1 and its pathophysiological effects on endothelial function. PAI-1 is produced by endothelial cells, hepatocytes, adipocytes, and macrophages and inhibits fibrinolysis by blocking tPA/uPA-mediated plasmin generation. Elevated PAI-1 contributes to impaired fibrin degradation, extracellular matrix accumulation, vascular inflammation, and endothelial dysfunction, ultimately promoting vascular stiffness and cardiometabolic risk. Red arrows indicate increased and decreased activity, while inhibitory bars indicate enzymatic inhibition.

PAI-1 has a half-life of approximately 5 minutes and is cleared by the liver. PAI-1 levels exhibit a distinct circadian variation, with plasma concentrations typically peaking in the early morning hours (17). This rhythmic pattern is thought to contribute to the morning increase in thrombotic cardiovascular events such as myocardial infarction and stroke (30). Circadian oscillations in PAI-1 expression are influenced by core clock genes and metabolic signals that regulate transcriptional activity in vascular, hepatic, and adipose tissues. It has been proposed that disruption of this circadian regulation exacerbates cardiometabolic risk and vascular dysfunction (20, 21, 30). In healthy individuals, plasma levels of PAI-1 generally vary from 5 to 20 ng/mL. However, these values increase with age and the presence of pathological conditions, with obesity, hyperinsulinemia, and age causing heightened PAI-1 expression in adipose tissue (24). Since this molecule’s discovery, its pathophysiological roles have been extensively studied in various human and animal disease models (14, 26). PAI-1 has been shown to be associated with cardiovascular diseases, metabolic disorders, aging, cancer, tissue fibrosis, inflammation, and neurodegenerative diseases (24). High circulating PAI-1 levels and activity are considered to be among the key mechanisms contributing to elevated cardiovascular risk in cases of obesity, and high PAI-1 levels also correlate positively with insulin resistance, obesity, and MetS (17, 22, 31, 32). Under normal conditions, PAI-1 production occurs among hepatocytes, platelets, adipocytes, vascular smooth muscle cells, fibroblasts, and macrophages/monocytes. In the presence of a pathological state, tumor cells and inflammatory cells can also produce PAI-1 (33). Gene expression databases indicate that PAI-1 expression is particularly enriched in adipose tissue, liver, and vascular tissues. Increased expression in visceral adipose depots and vascular compartments is consistent with its strong association with obesity-related cardiometabolic risk and vascular dysfunction (34). In addition to genetic variations, PAI-1 expression may be differentially regulated by body mass index (BMI), lipid/glucose metabolites, environmental factors (e.g., hypoxia, stress, and injuries), age, inflammation, and lifestyle (32). Among the genetic determinants of PAI-1 expression, the -675 4G/5G polymorphism located in the promoter region of the SERPINE1 gene has been particularly noteworthy. This insertion/deletion polymorphism affects the transcriptional regulation of the gene; the 4G allele is generally associated with higher transcriptional activity and increased circulating PAI-1 levels compared to the 5G allele (20, 21, 25). Recent studies suggest that this polymorphism may contribute to interindividual variability in thrombotic susceptibility, metabolic dysfunction, and cardiometabolic risk by modulating the basal and inducible expression of PAI-1 in metabolic and vascular tissues (20, 21, 25). Various chemical messengers, such as inflammatory cytokines, tumor necrosis factor-α (TNF-α), growth factors, hormones, and endotoxins, may also be responsible for changes in circulating PAI-1 levels (32). The cellular sources of PAI-1 and its pathophysiological effects on endothelial function are shown in **Figure 1**.

## 3 Structure, pathophysiology, and mechanisms of action of vaspin

Vaspin, a serpin protease inhibitor produced by visceral adipose tissue, is one of the most recently discovered adipokines and is categorized as member 12 of serpin class A (serpin A12) according to the serpin nomenclature (35). This adipokine plays an important role in obesity-related metabolic disorders (36). The *SERPINA12* gene encodes vaspin. This gene is located on the long arm of chromosome 14 (14q32.13) in humans and respectively comprises 1236, 1242, and 1245 nucleotides in rats, mice, and humans, synthesizing a vaspin protein of approximately 45 kDa (37, 38). Human vaspin comprises 395 amino acids and weighs 45.2 kDa. Vaspin expression has been observed in various human organs, including the skin, subcutaneous fat, the stomach, the pancreas, skeletal muscles, and the liver, and the highest levels of vaspin production occur in the liver (39). Gene expression databases demonstrate that vaspin is predominantly expressed in visceral adipose tissue, supporting its proposed role in adipose tissue function and metabolic regulation in obesity and insulin resistance (34).

The two protease targets of vaspin identified to date, kallikrein 7 (KLK7) and kallikrein 14 (KLK14), are inhibited by vaspin via a classical serpin mechanism (40). Members of the kallikrein family are involved in extracellular matrix remodeling, inflammatory signaling, and metabolic regulation in adipose tissue. Through the formation of stable serpin–protease complexes with KLK7 and KLK14, vaspin may regulate local proteolytic activity and modulate inflammatory responses in adipose and vascular tissues (40). In addition to its protease inhibitory activity, vaspin exerts several metabolic and cytoprotective effects through interaction with the 78-kDa glucose-regulated protein (GRP78), also known as heat shock protein family A member 5 (HSPA5) or binding immunoglobulin protein. GRP78 consists of three functional domains: a 44-kDa ATP-binding domain (ABD), a 20-kDa substrate-binding domain (SBD), and a 10-kDa C-terminal domain of currently unclear function (36). Under conditions of endoplasmic reticulum (ER) stress, GRP78 can translocate to the cell surface, where it acts as a binding partner for vaspin. Although GRP78 is frequently described as a functional receptor for vaspin, its precise role remains under debate, and it may function as a receptor-like signaling scaffold rather than a classical membrane receptor (41, 42). Binding of vaspin to cell-surface GRP78 has been reported to activate multiple intracellular signaling pathways, including PI3K/AKT, AMPK, NF-κB, and MAPK pathways, which collectively contribute to the anti-inflammatory, anti-apoptotic, and insulin-sensitizing effects attributed to vaspin. These signaling mechanisms are thought to play an important role in the protective effects of vaspin against metabolic dysfunction and vascular injury associated with ER stress (36–38, 43, 44).

Vaspin has been shown to be involved in various biological processes by interacting with numerous signaling mechanisms. It antagonizes TNF-α-induced NF-κB activation by accelerating AMPK phosphorylation. MCP-1 may protect vascular endothelial cells from TNF-α-induced damage by suppressing the production of cytokines such as intercellular adhesion molecule-1 (ICAM-1), E-selectin, and vascular cell adhesion molecule-1 (VCAM-1) (45). Upregulation of the phosphoinositide 3-kinase/Akt signaling pathway can protect vascular endothelial cells against free fatty acid-induced apoptosis. It can also protect endothelial cells against methylglyoxal-induced apoptosis by inhibiting caspase-3 activation and reducing nitrogen oxide (NOx)-induced ROS production (46). Vaspin has been reported to exert antiapoptotic effects on human aortic endothelial cells by binding to the GRP78/VDAC membrane complex, triggering AKT phosphorylation, and inhibiting Ca^2+^ influx (45, 47). Additionally, vaspin inhibits NF-κB/protein kinase C (PKC) and suppresses JAK2/STAT3 signaling pathway activity in smooth muscle cells (48).

**Figure 2:**
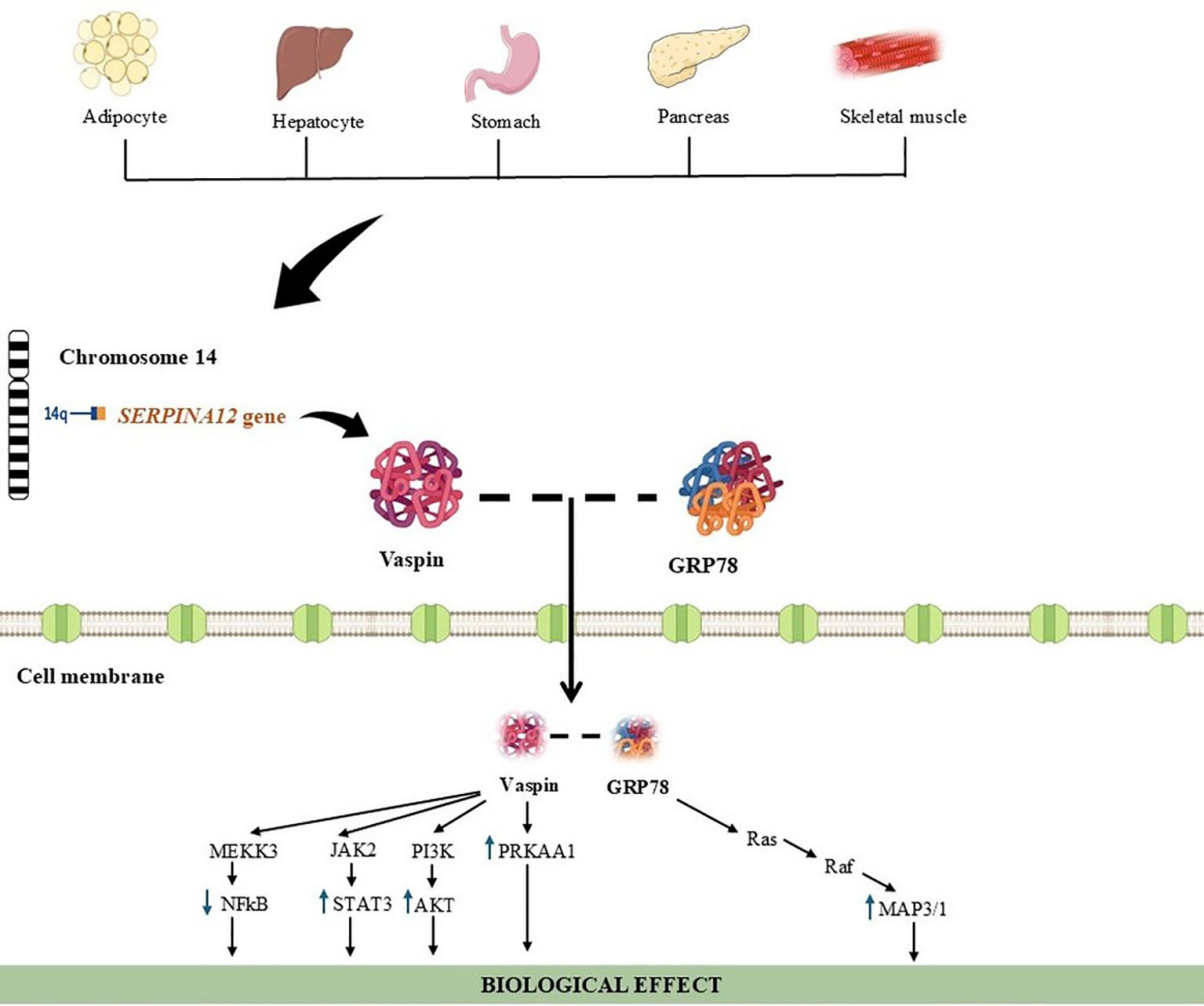
Origin, tissue expression, and mechanisms of action of vaspin. Vaspin, encoded by the SERPINA12 gene located on chromosome 14, is expressed predominantly in adipose tissue but is also detected in liver, stomach, pancreas, and skeletal muscle. After secretion, vaspin interacts with the cell-surface receptor GRP78, leading to activation or modulation of intracellular signaling pathways involved in metabolic regulation, inflammation control, and cell survival. These pathways include PI3K–AKT, JAK2–STAT3, PRKAA1, and Ras–Raf–MAPK signaling cascades, collectively contributing to improved metabolic homeostasis and anti-inflammatory effects (35, 36). MEKK3, Mitogen-activated protein kinase kinase kinase 3; JAK2, Janus kinase 2; PI3K, phosphoinositide 3-kinase; PRKAA1, protein kinase, AMP-activated, alpha 1; NFkB, nuclear factor kappa B; STAT3, signal transducer and activator of transcription 3; AKT, protein kinase B; MAP3/1, mitogen-activated protein kinase 3/1; ↑, increase; ↓, decrease; --: currently unknown.

The various effects of vaspin are capable of benefiting vascular health, appetite control, glucose metabolism, and lipid profiles (39). Vaspin expression levels in adipose tissue and plasma vaspin concentrations both increase at the peak of obesity and insulin resistance (49). This paradoxical tendency of vaspin to oppose adiponectin suggests a compensatory role for vaspin in cases of metabolic dysfunction (50). Vaspin protects β-cells from NF-κB-mediated inflammatory damage by promoting pancreatic islet cell secretion and reduces glucose production in the liver (51, 52). It is also known for its protective effects against insulin resistance and is found at increased levels in the presence of various metabolic disorders. Studies have shown that vaspin levels increase in conditions such as obesity, T2DM, and cardiovascular disease (39, 53). In addition, vaspin functions as a serine protease inhibitor, regulates glucose metabolism, and can reduce chronic inflammation thanks to its anti-inflammatory effects (54). The origin, tissue expression, and mechanisms of action of vaspin are shown in **Figure 2** (36, 37).

## 4 PAI-1 and vaspin in cardiometabolic diseases: molecular mechanisms and clinical implications

Adipose tissue is a crucial endocrine organ secreting a variety of adipokines that regulate inflammation (55). In individuals with obesity, adipose tissues produce excessive levels of proinflammatory cytokines including TNF-α and interleukin (IL)-18, IL-1β, and IL-8, all of which promote inflammation (55, 56). Elevated plasma PAI-1 levels are seen in individuals with obesity and increase the likelihood of the occurrence of MetS, T2DM, and cardiovascular diseases. Increased PAI-1 expression in adipose tissue in cases of obesity occurs through the TNFα-TNFR (P55/P75) signaling pathway (19, 57). In contrast, the inhibition of PAI-1 expression in mice lowers blood glucose and prevents inflammation, most likely by reducing TNF-α expression (58).

Pharmacologically inhibiting plasma PAI-1 in animal models resulted in body weight loss and reductions in adipose tissue and adipocyte volumes (16). In the study conducted by Somodi et al. (2018), PAI-1 concentrations were investigated in non-diabetic individuals with obesity and significantly higher PAI-1 levels were found in the obese group compared to lean controls. In another study, it was found that there was an increase in plasma PAI-1 in both patients with T2DM and healthy individuals with obesity (59). However, no significant difference was observed in the genotype distribution of the PAI-1 4G/5G polymorphism in non-diabetic individuals with obesity compared to a control group with normal body weight (60). A population-based study suggested higher circulating PAI-1 levels and low adiponectin expression as possible predictors of MetS (61). Inhibition of PAI-1 supports body weight loss in cases of obesity by upregulating the expression of phosphorylated perilipin-1 and lipases, thus favoring adipose tissue lipolysis (62).

**Figure 3:**
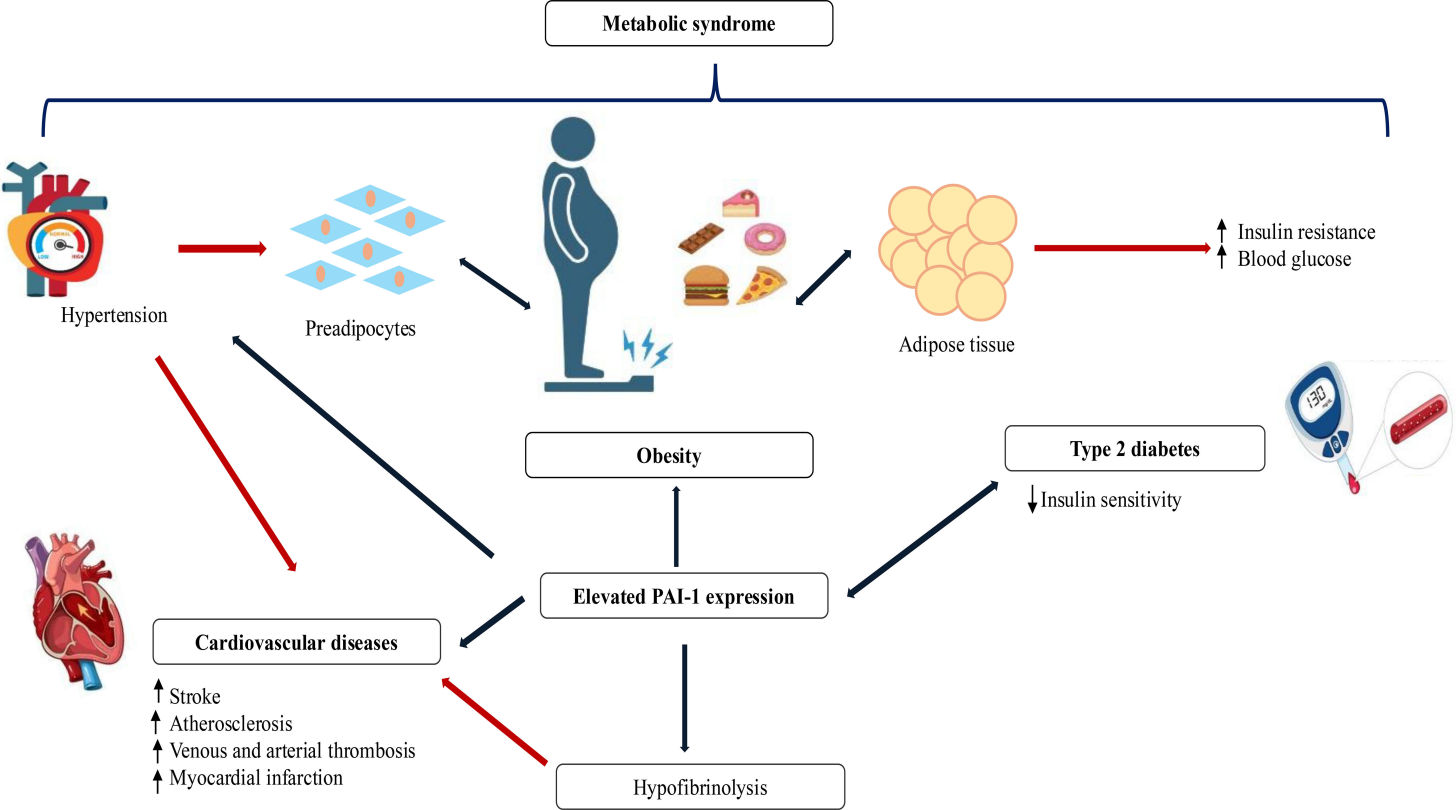
Role of PAI-1 in the development of cardiometabolic diseases. Elevated PAI-1 expression represents a key mechanistic link between obesity, metabolic syndrome, and type 2 diabetes and the development of cardiovascular complications. Expansion of adipose tissue and associated metabolic disturbances promote increased PAI-1 production, leading to hypofibrinolysis and vascular dysfunction. These alterations facilitate thrombosis, atherosclerosis, and heightened cardiovascular risk, while reciprocal interactions with insulin resistance, hypertension, and obesity further amplify cardiometabolic disease progression (22). Blue arrows represent (patho-)physiological processes that arise as a result of increased PAI-1 levels and red arrows illustrate the links between each of these individual pathologies; ↑: increased; ↓: decreased.

The role of PAI-1 in vascular pathology is supported by protein accumulation in areas of vascular atheroma, which appears to be particularly prominent in diabetic individuals (22). It was reported that high soluble thrombomodulin in diabetes is an indicator of ongoing damage caused by hyperglycemia, and high plasma concentrations of soluble thrombomodulin in patients with T2DM may cause destructive effects such as widespread vascular damage (63). It is hypothesized that PAI-1 may play a role in the pathogenesis of venous thromboembolism (VTE), particularly obesity-associated VTE (64). One study found that the risk of VTE increased dose-dependently among PAI-1 tertiles according to age and gender (64). In the same study, PAI-1 mediated approximately 15% of the VTE risk in cases of obesity, as reflected by C-reactive protein levels, and this finding was generally independent of chronic low-grade inflammation. PAI-1 might be a future target for interventions to reduce the risk of VTE in individuals with obesity (64). PAI-1 and VCAM-1 have been reported to be significantly increased in diabetic vascular disorders (65). Therefore, lowering patients’ blood glucose may help in preventing diabetic cardiorenal complications by inhibiting inflammation (65, 66). Considering the relationship of PAI-1 with the renin-angiotensin system and the increases in PAI-1 levels observed in the presence of hypertension, the mechanisms supporting this relationship need to be investigated in more detail (65, 67). The proposed role of PAI-1 in the occurrence and progression of cardiometabolic diseases is shown in **Figure 3** (22).

Vaspin is a novel adipokine currently attracting attention for its potential role in metabolic regulation, particularly in relation to T2DM (68). It is predominantly upregulated in response to increased insulin resistance, suggesting a compensatory mechanism to counteract that resistance in individuals with obesity and MetS (68, 69). Vaspin levels in both granulosa cells and follicular fluid were higher in obese women, positively correlating with BMI values (70), while another study found that obese children with MetS had higher vaspin concentrations than obese children without MetS (71). Vaspin may exert various protective effects that help improve the components of MetS (69). Additionally, it has anti-inflammatory properties capable of reducing chronic inflammation and it supports endothelial function, thus frequently benefiting cardiovascular health in individuals with MetS (69, 72).

**Figure 4:**
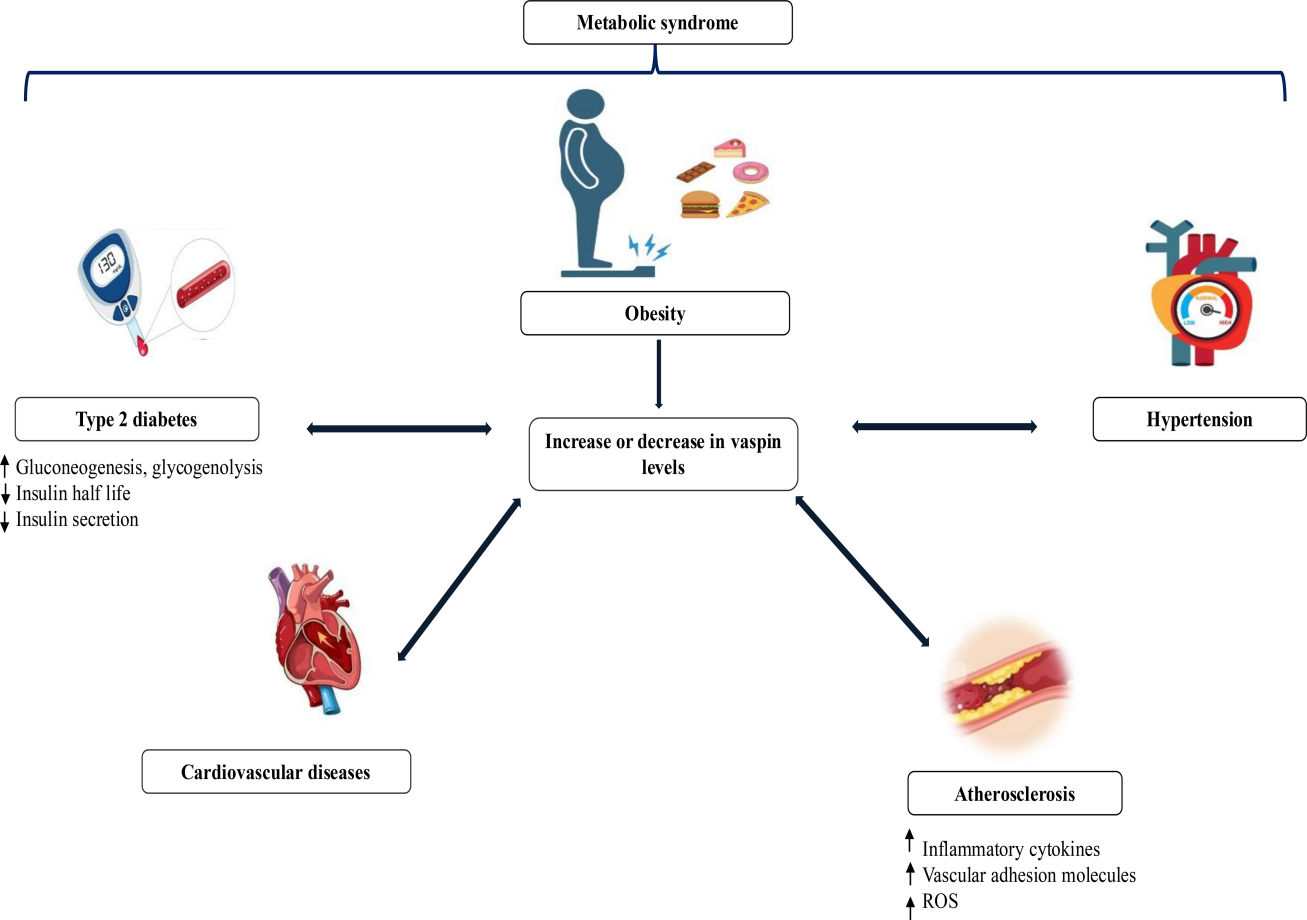
Role of vaspin in the development of cardiometabolic diseases. Alterations in circulating vaspin levels are closely associated with obesity and metabolic syndrome and influence the development of type 2 diabetes, hypertension, atherosclerosis, and cardiovascular diseases. Changes in vaspin concentrations are linked to disturbances in insulin secretion and action, inflammatory activity, oxidative stress, and vascular dysfunction, thereby contributing to cardiometabolic disease progression and vascular complications (13, 78). ROS, Reactive oxygen species; ↑, increased; ↓, decreased.

Administration of vaspin in animal models results in improved glucose tolerance and insulin sensitivity, which are crucial in the management and prevention of T2DM (10). In one study, the intraperitoneal injection of vaspin into rats resulted in the improvement of insulin resistance within adipose tissues, the liver, and skeletal muscles through increased transduction of the IRS/PI3K/Akt/GLUT2 pathway while also inhibiting the IκBα/NFκB signaling pathway, consistent with results from experiments involving rats fed high-fat diets (73). In the study conducted by Özkan et al. (2022), vaspin levels were shown to be significantly lower in overweight and obese children with nonalcoholic fatty liver disease and were positively correlated with high-density lipoprotein cholesterol. In another study, serum vaspin was found to be significantly lower in patients with T2DM compared to a control group (74). In contrast, Zieger et al. (2018) found no effect of vaspin in their study. Other studies have shown that vaspin, due to its insulin sensitivity-enhancing and anti-inflammatory effects, has a protective mechanism in obesity, which may be described as chronic inflammation due to insulin resistance (36, 52). There are limited and inconsistent clinical data on the relationship between vaspin levels and cardiovascular disease (67). Sato et al. (2018) reported that vaspin is antiatherosclerotic and improves plaque stability in apoE −/− mice. In a large cohort of patients with axial spondylarthritis, serum vaspin was associated with risk factors for cardiovascular disease (75). A study of individuals experiencing chest pain indicated the utility of vaspin as a predictive biomarker of major adverse cardiovascular events (76). Vaspin expression levels significantly increased the risk of ischemic stroke in a prospective study of individuals diagnosed with T2DM (77). In one study, vaspin successfully suppressed the proliferation and migration of vascular smooth muscle cells via the downregulation of the ERK1/2 and c-Jun N-terminal kinase pathways and increased collagen production via the upregulation of PI3K/Akt pathways (50). However, additional studies are still necessary to clarify the possible contributions of vaspin as a therapeutic agent or biomarker for atherosclerotic cardiovascular diseases. The role of vaspin in the development of cardiometabolic diseases is shown in **Figure 4** (13, 78) and the regulatory roles, mechanisms, and biomarker potential of PAI-1 and vaspin in cardiometabolic disorders are presented in **Table 1**. There are numerous clinical studies in the literature supporting the relationship of these biomarkers with metabolic disorders, as summarized in **Table 2**.

**Table 1:** Functional roles of adipokines PAI-1 and vaspin in cardiometabolic disorders with a focus on regulation, mechanisms, and biomarker potential.

Adipokine	Change	Metabolic effects	References
PAI-1	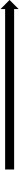	Decreased insulin sensitivity Thrombotic effects and inflammation Increased liver steatosis and serum cholesterol Increased atherogenesis with macrophage accumulation Associations with markers of atherogenic dyslipidemia (correlations with HDL subfractions and ApoAI; PAI-1 release stimulated by small-sized HDL in adipocytes; VLDL capable of increasing PAI-1 levels in endothelial cells) Biomarker for obesity, insulin resistance, and T2DM	(79–82)
Vaspin	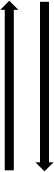	Insulin-sensitizing and anti-inflammatory agent via a compensatory mechanism Involvement in hypertension Involvement in lipid metabolism Improves glucose tolerance via enhanced insulin sensitivity Reduces β-cell apoptosis and supports insulin secretion Modulates ER stress via GRP78 interaction protease inhibition Biomarker for obesity, T2DM, and NAFLD	(74, 83–85)

↑, Increased; ↓, decreased; ApoAI, apolipoprotein AI; ER, endoplasmic reticulum; HDL, high-density lipoprotein; LDL, low-density lipoprotein; NAFLD, nonalcoholic fatty liver disease; PAI-1, plasminogen-activator inhibitor-1; T2DM, type 2 diabetes mellitus; VLDL, very low-density lipoprotein.

**Table 2:** Relationships of PAI-1 and vaspin levels with cardiometabolic parameters in human studies.

	Study design	Participants	Mean age (years)	Male (%); female (%)	Ethnicity	Outcomes or parameters	Direction of relationship with cardiometabolic health*	References
PAI-1								
	CS	60 newly diagnosed T2DM patients	NR	NR	Asian	T2DM, arterial stiffness	Negative	(86)
	CS	60 with MetS, 142 without MetS	45.8 (MetS), 39.7 (controls)	66.4; 33.6	NR	MetS	Negative	(31)
	PCC	60 T2DM patients, 30 healthy controls	NR	NR	Black	T2DM and CAD	Negative	(87)
	CS	80 participants (40 NAFLD patients, 40 controls)	43.3 (NAFLD patients), 46.3 (controls)	50; 50	Asian	NAFLD	Negative	(88)
	CS	451 (obese pre-MetS and MetS patients)	NR	NR	NR	MetS	Negative	(89)
	CC	196 T2DM patients and 50 healthy controls	NR	NR	White	Type 2 diabetic nephropathy	Negative	(90)
	CS	47 women	40.0	100.0	NR	Obesity	Negative	(91)
	RC	130 (65 with MetS, 65 controls)	52.2	54.6; 45.4	White	MetS	Negative	(92)
	CS	164 MetS patients, 100 controls	42.7	53.8; 46.2	Asian	Carotid intima-media thickness, MetS	Negative	(93)
	PCC	484 new cases of T2DM	48.8	47.9; 52.1	NR	Systolic and diastolic blood pressure Fasting plasma glucose Total cholesterol HDL cholesterol Triglycerides Physical activity	Negative	(94)
Vaspin								
	CS	121 obese children	NR	34.7; 65.3	Asian	MetS	Negative	(71)
	RC	474 patients with NAFLD	NR	NR	Asian	Exercise	Negative	(95)
	RC	110 patients with ACS	48.6	65.5; 34.5	Asian	Rosuvastatin	Positive	(96)
	CS	70 women	29.0	0; 100	Asian	Obesity	Negative	(97)
	PC	85 patients with CAD	64.0	77.6; 22.4	White	In-stent restenosis	Positive	(98)
	CC	120 T2DM patients, 120 controls	NR	NR	South Asian	T2DM	Negative	(99)
	RC	197 individuals with chest pain	65.0	56.9; 43.1	Asian	Major adverse cardiovascular event	Positive	(76)
	PCC	90 patients with T2DM	58.7	55.6; 44.4	Asian	Ischemic stroke	Negative	(77)
	CS	372 patients with T2DM	53.0	55.6; 44.4	Asian	Diabetic retinopathy	Negative	(100)
	NRI	84 individuals with preclinical carotid atherosclerosis	62.0	46.4; 53.6	White	Atorvastatin	Positive	(101)
	CS	114 patients in total (31 T2DM, 23 chronic pancreatitis, 60 T2DM + chronic pancreatitis); 20 healthy controls	NR	36.8; 63.2	NR	T2DM and chronic pancreatitis	Positive	(102)
	CC	90 participants (60 with MetS, 30 controls)	NR	NR	Iraqi/Arab	MetS	Negative	(103)
	CS	40 obese patients and 20 normal-weight subjects	43.3 (obsese), 38.9 (normal weight)	50.0; 50.0	Caucasian	Obesity	Positive	(104)
	CC	90 participants (30 healthy controls, 30 with T2DM, 30 with T2DM + diabetic nephropathy)	75.0	50.0; 50.0	African	T2DM and diabetic nephropathy	Negative	(105)

NS, Not specified; NR, not reported; RC, randomized controlled trial; NRI, nonrandomized intervention; PC, prospective cohort; RC, retrospective cohort; PCC, prospective case-control; CS, cross-sectional; ROS, retrospective observational study; T2DM, type 2 diabetes mellitus; MetS, metabolic syndrome; NAFLD, nonalcoholic fatty liver disease; ACS, acute coronary syndrome; CAD, coronary artery disease, HDL, high-density lipoprotein.

*Positive relationship: Increasing adipokine levels are associated with improved cardiometabolic health; negative relationship: increasing adipokine levels are associated with decreased cardiometabolic health.

### 4.1 Sex-specific regulation and hormonal influences on PAI-1 and vaspin

Increasing evidence suggests that the regulation of adipokines such as PAI-1 and vaspin may be influenced by sex hormones and reproductive status (56, 67). (Estrogens have been reported to modulate fibrinolytic activity and may contribute to lower PAI-1 levels in premenopausal women compared with men, whereas androgen excess may alter adipokine secretion and metabolic risk profiles (31, 32). In addition, vaspin has been investigated in reproductive disorders such as polycystic ovary syndrome (PCOS), where altered adipokine levels have been observed in ovarian tissues and follicular fluid (53, 70). These findings indicate that hormonal status and sex-specific physiological differences may influence adipokine regulation and could partly explain variations in cardiometabolic risk between men and women (56, 68). However, further research is needed to clarify the underlying molecular mechanisms linking sex hormones, adipokine regulation, and cardiometabolic disease development.

## 5 Regulatory effects of dietary and lifestyle factors on PAI-1 and vaspin

An inflammatory diet refers to a dietary pattern characterized by high intake of refined carbohydrates, saturated and trans fatty acids, processed meats, and sugar-sweetened beverages, which may promote chronic low-grade inflammation by increasing production of pro-inflammatory cytokines and oxidative stress. In contrast, anti-inflammatory diets are typically rich in fruits, vegetables, whole grains, legumes, nuts, fish, and unsaturated fatty acids, and are associated with lower levels of inflammatory biomarkers (106). Evidence from human studies has demonstrated that diets with a higher inflammatory potential, often assessed using the Dietary Inflammatory Index (DII), are associated with increased circulating inflammatory markers such as C-reactive protein (CRP) and interleukin-6 (IL-6), whereas adherence to anti-inflammatory dietary patterns such as the Mediterranean diet has been linked to reduced systemic inflammation and improved cardiometabolic profiles (107–109).

A proinflammatory diet predisposes to the development of components of cardiometabolic disease such as insulin resistance, hypertension, dyslipidemia, and hyperglycemia. Conversely, an anti-inflammatory diet reduces the risk of developing chronic diseases and prevents cardiometabolic disease (110). In a study conducted with obese women, it was observed that dietary inflammatory index scores and PAI-1 levels increased in those who consumed foods with high energy contents (111). In a study comparing individuals with MetS and healthy individuals, PAI-1 levels improved when dietary polyunsaturated fatty acid (PUFA) contents were replaced with monounsaturated fatty acid (MUFA) contents. However, no significant difference in PAI-1 levels was observed in healthy individuals (112). Another study showed that replacing dietary saturated fatty acid with MUFA or dietary carbohydrates in individuals with MetS had no effect on PAI-1, suggesting the need for further research in individuals with MetS (113).

Diets high in antioxidant capacity may reduce levels of PAI-1, a key regulator of thrombotic processes. For example, Radkhah et al. (2022) reported significantly lower PAI-1 concentrations in individuals consuming diets with high antioxidant capacity. Similarly, the study conducted by Shiraseb et al. (2023) revealed that increased dietary polyphenol intake correlated with decreased PAI-1 levels. Considering that PAI-1 possesses a critical role in the processes of endothelial dysfunction, fibrinolysis inhibition, and the development of atherosclerosis, these findings support the claim that a diet rich in antioxidants may be a protective strategy in reducing the risk of cardiovascular disease (114, 115).

Prevention strategies for cardiometabolic diseases include a focus on modifiable risk factors such as diet and physical activity (116). Moderate-to-vigorous physical activity of ≥150 minutes per week has been shown to significantly reduce PAI-1 levels in individuals with diabetes as well as in individuals without diabetes (116). It is suggested that aerobic physical activity may have a greater effect on PAI-1 levels than resistance training in an intensity-dependent manner, with physical activity of higher intensity being associated with greater reductions in PAI-1 levels (28). Higher levels of physical activity have also been associated with lower PAI-1 levels in individuals at risk for cardiometabolic disease (117). This suggests that regular physical activity is a potential early intervention tool in this population.

As previously discussed, vaspin suppresses oxidative stress and inflammation by exerting anti-inflammatory effects, and thanks to these properties, it may play a potential protective role in the prevention of cardiometabolic diseases (118). Improvement in vaspin levels was observed in individuals with obesity who adopted a Mediterranean diet (119). Similarly, body weight loss and a Mediterranean diet reduced vaspin levels in individuals with T2DM (120). Vaspin levels can be regulated by physical activity as well as nutrition. One study found that a healthy diet and regular physical activity increased vaspin concentrations in slightly overweight and obese participants (121). Similarly, aerobic exercise and resistance exercise have been shown to be associated with changes in vaspin levels and endothelial function in individuals with T2DM (122). These findings suggest that circulating vaspin levels are related to vascular function during lifestyle changes in T2DM.

In addition to facilitating body weight loss, bariatric surgery reduces the risks and complications of T2DM, hypertension, dyslipidemia, cardiovascular disease, and nonalcoholic fatty liver disease (123, 124). One study found that Dietary Inflammatory Index and Energy-Adjusted Dietary Inflammatory Index scores improved and PAI-1 levels decreased after bariatric surgery. Decreases in total energy, carbohydrate, protein, lipid, dietary cholesterol, saturated fat, MUFA, PUFA, and sodium intake were also observed (125). In another similar study, a decrease in PAI-1 levels was observed along with a decrease in inflammatory markers after bariatric surgery (126). Vaspin levels have also been assessed following bariatric surgery. For example, after bariatric surgery, individuals’ vaspin levels were observed to gradually decrease over the course of 12 months (127). Such findings suggest that lifestyle factors such as diet composition, physical activity level, and surgical interventions could significantly influence PAI-1 and vaspin levels, potentially playing regulatory or protective roles in the pathophysiology of cardiometabolic diseases. However, further research, including long-term human studies, is needed to better understand these mechanisms.

## 6 Therapeutic potential of PAI-1 and vaspin

PAI-1 and vaspin have attracted attention as therapeutic targets due to their roles in various chronic diseases and intensive research has begun on drugs and vaspin-based pharmacological agents that can modulate PAI-1 activity (14). Potential inhibitors including low-molecular-weight molecules, peptides, monoclonal antibodies, and antibody fragments have been extensively studied (14, 22). While some PAI-1 inhibitors are currently under investigation for conditions besides obesity, none have received approval for clinical use to date. New insights into the biology of PAI-1 suggest that the indirect targeting of PAI-1 with regulatory pathways or inhibitors may yield promising new therapeutic options for reducing the risk of VTE in cases of obesity (22, 64). PAI-1 inhibitor therapy may also protect against high-fat diet-induced obesity, insulin resistance, and fatty liver disease, in part through attenuated hypothalamic leptin resistance and increased energy expenditure associated with increased adipocyte lipolysis (62, 128). For example, obese mice that were administered a PAI-1 inhibitor showed significant reductions in macrophage infiltration within white adipose tissues and heightened levels of anti-inflammatory M2 polarization (16). More research will be needed to clarify the molecular mechanisms by which PAI-1 impacts the processes of macrophage polarization, but current evidence implies that targeting PAI-1 may produce new therapeutic strategies for addressing obesity and MetS (22).

Vaspin is also attracting attention as a possible therapeutic target, particularly in metabolic diseases characterized by insulin resistance, obesity, and inflammation (68). Thanks to its ability to inhibit serine proteases, vaspin appears to increase insulin sensitivity and modulate cellular stress responses. Additionally, measuring vaspin levels can aid in the early diagnosis and monitoring of MetS, enabling timely and targeted interventions (129). Preliminary trials with animal models have been promising, showing that vaspin administration improves glucose tolerance and alleviates endoplasmic reticulum stress (36, 130). It is also reported to have antiatherogenic effects on the cardiovascular system, support vascular endothelial function, and suppress inflammatory responses (75). However, further research is needed to determine the effectiveness and safety of vaspin-based treatments in humans (131). In the context of next-generation biologic drugs and gene therapies, recombinant forms of vaspin or compounds that modulate vaspin levels are being evaluated as potential candidates for the treatment of diabetes, atherosclerosis, and even some neurological diseases (37), but human-based studies and long-term safety analyses are still needed for a transition to clinical practice.

## 7 Conclusions

The activities of adipokines PAI-1 and vaspin are linked to T2DM, cardiovascular disease, obesity, and MetS. Given the metabolic effects of these molecules, their functions in the pathogenesis of these diseases are quite complex. Discrepant results are observed in the literature for both PAI-1 and vaspin. Both proinflammatory and anti-inflammatory effects have been reported for the same adipokines. This suggests that their biological effects may depend on the specific inflammatory context and disease state.

When the effects of dietary and lifestyle factors on PAI-1 and vaspin are evaluated together, it appears that these two adipokines are critical regulators in both the development and management of cardiometabolic diseases. Polyphenol-rich diets, Mediterranean-style diets, and high antioxidant capacity significantly reduce PAI-1 levels, providing vascular protection. Similarly, regular physical activity, including both aerobic and resistance exercise, positively affects both PAI-1 and vaspin levels, improving the metabolic risk profile. In conclusion, PAI-1-targeting inhibitors and vaspin-based biologic agents stand out as future therapeutic candidates for the treatment of diseases such as diabetes, atherosclerosis, and MetS. Lifestyle interventions, including improved nutritional quality, regular physical activity, body weight management, and bariatric surgery, also appear to offer natural and effective regulatory mechanisms for these biomarkers.

To more fully understand these two adipokines and their potential prognostic and diagnostic value in the treatment of obesity-related disorders such as MetS, T2DM, and cardiovascular diseases, more cell line, *in vivo, in vitro*, animal, and human clinical studies and experimental research are necessary. Such research may allow the clarification of the contributions of these adipokines and would support more reliable generalizations.

## 8 Future perspectives

Despite the current recognition of the prominent roles of PAI-1 and vaspin in the pathogenesis of MetS, obesity, cardiovascular disease, and T2DM, more comprehensive human studies are necessary for a more thorough understanding of the clinical applications of these adipokines. Future research evaluating the efficacy and safety of small molecule inhibitors and vaspin-based biologic agents targeting PAI-1 will further delineate the potential of these two adipokines as therapeutic targets. Furthermore, long-term clinical studies, particularly in humans, are expected to confirm the value of PAI-1 and vaspin as biomarkers predicting disease progression. Studies addressing the regulatory effects of diet quality, lifestyle interventions, and genetic variations on these adipokines will contribute to the development of new approaches for both early diagnosis and personalized treatment strategies.
